# Magnetic resonance imaging detection of avascular necrosis in carpal bones despite apparent fracture union: a case series

**DOI:** 10.11604/pamj.2026.54.18.53032

**Published:** 2026-05-25

**Authors:** Santosh Kumar Panda, Madhuri Panigrahi, Birajman Lakra

**Affiliations:** 1 Department of Radiodiagnosis, Dharanidhar Medical College, Keonjhar, Odisha, 758001, India,; 2 Department of Physiology, Srirama Chandra Bhanja Medical College, Cuttack, Odisha, 753007, India

**Keywords:** Magnetic resonance imaging, avascular necrosis, scaphoid, lunate, wrist injuries

## Abstract

Assessment of fracture healing in carpal bones is commonly performed using computed tomography (CT). However, CT may not accurately reflect bone viability. Magnetic resonance imaging (MRI) is more sensitive in detecting early changes in bone marrow and vascularity. A diagnostic challenge arises when CT suggests fracture union while MRI demonstrates features of avascular necrosis (AVN). We conducted a retrospective case series of three patients with prior carpal bone fractures (one scaphoid and two lunate) who presented with persistent or new-onset wrist pain following apparent fracture healing. All patients underwent MRI evaluation, and a CT was performed in one case for correlation. Magnetic resonance imaging (MRI) demonstrated features suggestive of AVN in all three cases despite prior imaging indicating fracture union. Two cases showed T1 hypointensity with corresponding hyperintensity on fluid-sensitive sequences, consistent with early AVN, while one case demonstrated persistent low signal on both sequences, suggestive of chronic AVN. In the case of CT correlation, there was apparent fracture union with mild sclerosis and no evidence of collapse, highlighting discordance between structural and biological assessment. Hence, persistent wrist pain following a healed fracture should prompt MRI evaluation to assess bone viability and guide appropriate management.

## Introduction

Fractures of the carpal bones are common injuries of the wrist. They are associated with a risk of complications, including nonunion and avascular necrosis (AVN) [1,2]. The scaphoid is the most frequently fractured carpal bone and is prone to AVN. This is due to its retrograde blood supply mechanism, rendering the proximal pole susceptible to ischemia following fracture [2,3]. The lunate has a tenuous vascular supply and is susceptible to AVN, which may occur idiopathically or following trauma [4]. Radiological assessment of fracture healing relies on computed tomography (CT) [5]. However, CT primarily evaluates structural integrity and may not accurately reflect the biological viability of bone. In contrast, magnetic resonance imaging (MRI) is more sensitive in detecting early changes in bone marrow. This feature of MRI helps in identification of ischemia and AVN [6,7]. In some cases, CT and MRI findings may not align, with CT suggesting fracture union while MRI indicates possible avascular necrosis. This highlights a limitation of structural imaging and the need for further assessment of bone viability in patients who remain symptomatic [7,8]. In this report, we describe three patients with prior carpal bone fractures-one involving the scaphoid and two involving the lunate-in whom MRI demonstrated features suggestive of avascular necrosis despite apparent fracture healing on earlier imaging.

## Methods

**Study design and patients:** this retrospective case series was conducted at a tertiary care center in Odisha, India, over a study period from November 2025 to March 2026. It consists of three patients presenting with wrist pain following previously diagnosed and conservatively managed carpal bone fractures. All patients had prior X-ray imaging suggesting radiological fracture union and had resumed routine activities.

**Inclusion criteria:** patients were included based on the following criteria: history of prior carpal bone fracture; apparent fracture healing on prior imaging; persistent or recent-onset wrist pain following presumed union.

**Exclusion criteria:** known inflammatory arthritis or degenerative joint disease; history of wrist surgery; inadequate imaging or incomplete clinical data.

**Imaging technique:** magnetic resonance imaging of the wrist was performed using standard protocols (axial, coronal and sagittal planes), including T1- and T2-weighted sequences; fluid-sensitive sequence (proton density fat-saturated). Images were evaluated for marrow signal abnormalities; evidence of prior fracture sites; signs of avascular necrosis; carpal alignment and associated soft tissue changes. In one case, computed tomography (CT) of the wrist was performed for correlation, primarily to assess: fracture union; cortical integrity; presence of sclerosis or collapse.

**Image analysis:** all imaging studies were reviewed descriptively by experienced radiologists.

**Ethical consideration:** ethical approval was waived due to the retrospective nature of the study. Written informed consent was obtained from all patients for publication.

## Results

A total of three patients with a prior history of conservatively managed carpal bone fractures were included in our case series. All patients had imaging evidence suggestive of fracture union on earlier radiographs and had resumed routine activities. However, they subsequently presented with persistent or new-onset wrist pain, prompting further evaluation with MRI. In all cases, MRI demonstrated focal marrow signal abnormalities suggestive of avascular necrosis, despite the absence of structural collapse or malalignment. These findings highlight the discrepancy between apparent fracture healing and underlying bone viability. The clinical and imaging findings of all three cases are summarized in Table 1, demonstrating a spectrum of avascular necrosis ranging from early to chronic stages despite apparent fracture union.

**Table 1:** clinical and imaging characteristics of patients with suspected post-traumatic avascular necrosis despite apparent fracture union

Parameter	Case 1	Case 2	Case 3
Age (in years)	25	32	49
Sex	Male	Female	Male
Bone involved	Scaphoid (proximal pole)	Lunate	Lunate
History of trauma	Yes (9 months prior)	Yes (few years prior)	Yes (few years prior)
Initial management	Conservative	Conservative	Conservative
Status before presentation	Radiological union, asymptomatic period	Radiological union	Radiological union
Presenting symptom	Recent-onset wrist pain	Persistent wrist pain	Mild chronic wrist pain
T1 signal	Hypointense (proximal pole)	Hypointense (focal)	Hypointense
Fluid-sensitive signal (PD-FS)	Hyperintense (marrow edema)	Hyperintense	Hypointense
Associated findings	Waist edema, prior fracture line. No malalignment	No malalignment	No malalignment
CT findings	Union with proximal pole sclerosis	Not performed	Not performed
Stage of AVN (MRI-based)	Early AVN	Early AVN	Chronic AVN
Collapse	No	No	No
Contrast MRI	Not done	Not done	Not done
Follow-up	Not available	Not available	Not available

MRI: magnetic resonance imaging, AVN: avascular necrosis

**Figure 1:**
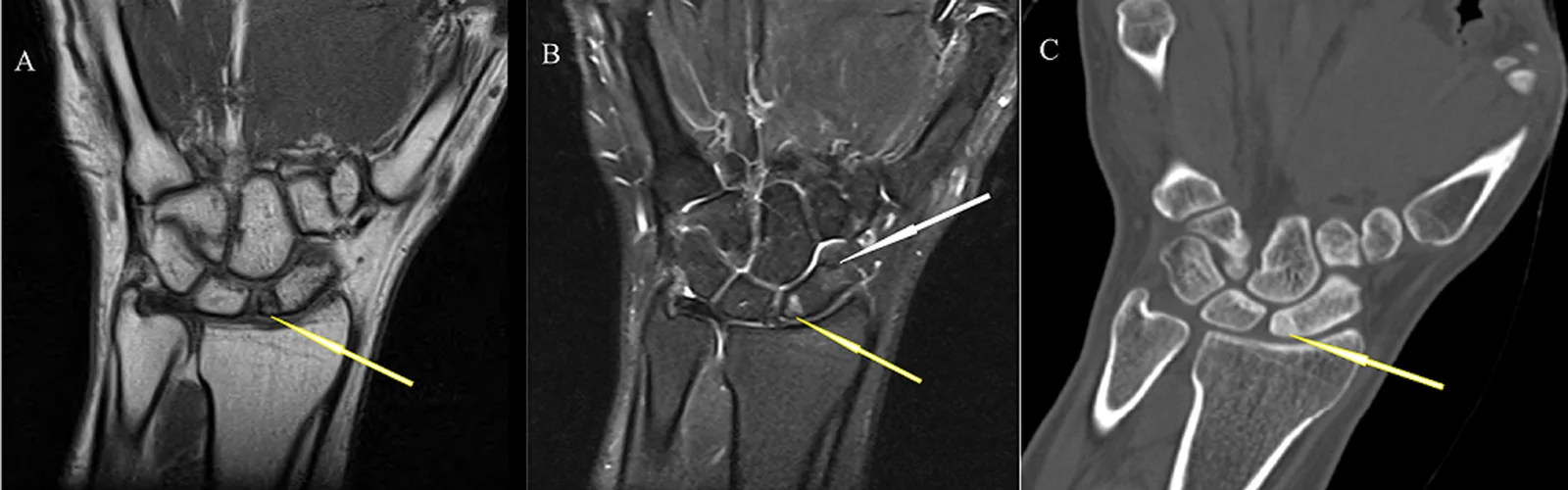
A) coronal T1-weighted MRI of the wrist shows a focal hypointense marrow signal abnormality in the proximal pole of the scaphoid (yellow arrow), suggestive of early ischemic change; B) coronal proton density fat-saturated MRI demonstrates a linear hypointense signal at the scaphoid waist (white arrow), consistent with a prior fracture site, with associated marrow edema; a focal hyperintense signal in the proximal pole (yellow arrow) raises suspicion for early avascular necrosis; C) coronal CT image shows apparent union of the scaphoid waist fracture with focal sclerosis of the proximal pole and no visible fracture line or collapse; these findings indicate structural healing, with sclerosis that may correlate with MRI-detected early vascular compromise

**Case 1:** a 25-year-old male with a history of right wrist scaphoid fracture 9 months prior, managed conservatively, presented with persistent wrist pain of recent onset (for the last few days). The fracture had been previously considered radiologically healed, and the patient had resumed routine activities. Blood test reports were normal. Magnetic resonance imaging of the wrist was performed for further evaluation. Magnetic resonance imaging revealed a 5 x 4 mm focal lesion in the proximal pole of the scaphoid, showing heterogeneous hypointensity on T1-weighted images and hyperintensity on fat-suppressed sequences (Figure 1A, B). These findings were suggestive of early avascular necrosis (AVN), with possible residual viable bone. Marrow edema was also noted in the scaphoid waist region, corresponding to the prior fracture site. No carpal malalignment was identified. No adjacent fluid collection was seen. The adjacent soft tissue did not show any abnormal signal change. No feature of degenerative joint disease or arthritis was seen. A complementary CT scan was performed to better correlate the MRI findings, and CT images revealed: apparent fracture union and mild sclerosis of the proximal pole of the scaphoid bone (Figure 1C). No evidence of collapse. No additional abnormalities were detected.

**Figure 2:**
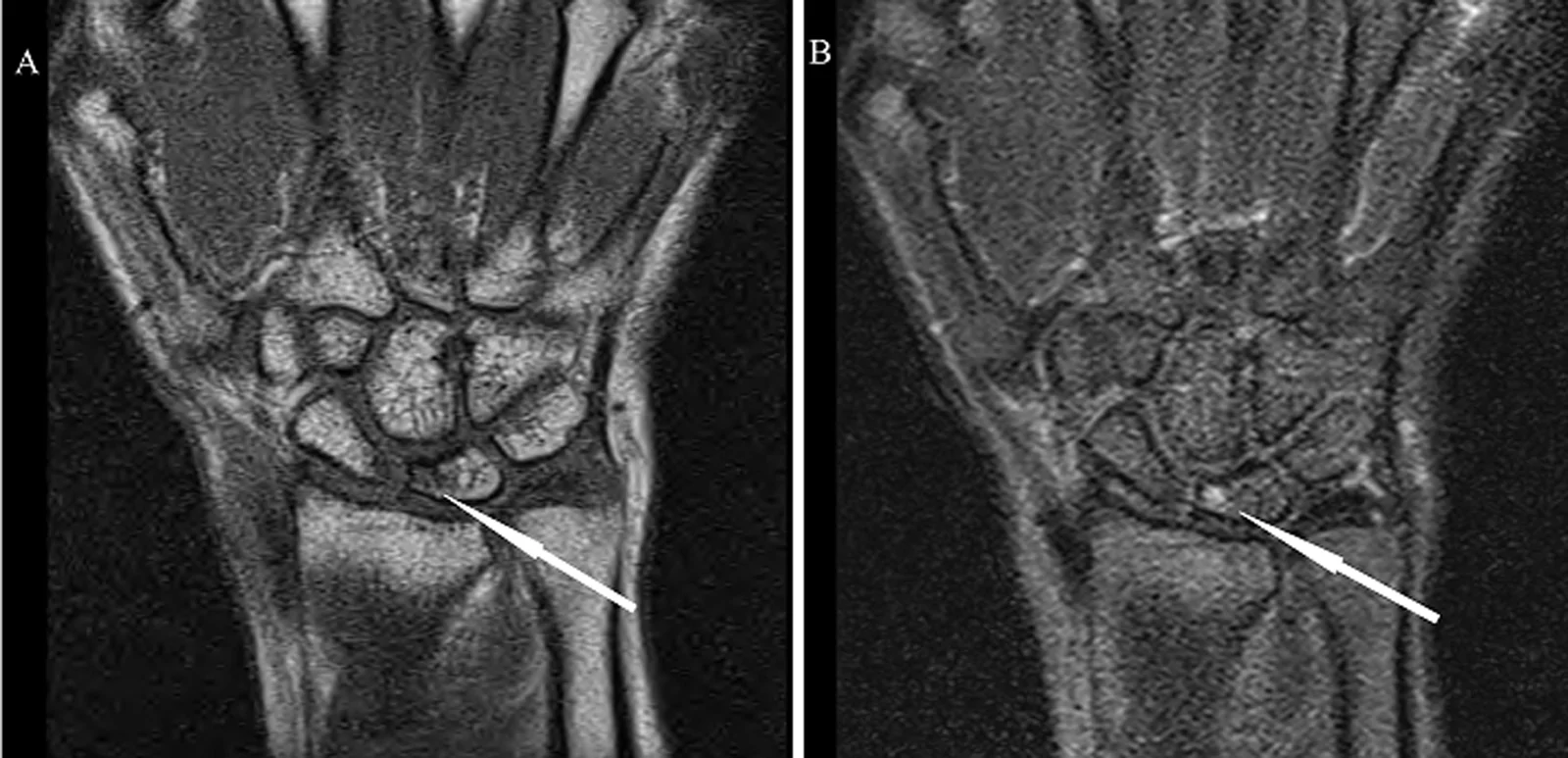
A) coronal T1-weighted MRI of the wrist demonstrates a focal hypointense marrow signal in the lunate (arrow), suggestive of early ischemic change; B) coronal proton density fat-saturated (PD-FS) MRI shows a corresponding focal hyperintense signal in the lunate (arrow), consistent with marrow edema, raising suspicion for early avascular necrosis

**Case 2:** a 32-year-old female presented with a history of a left wrist lunate fracture a few years prior. She was managed conservatively. For the last few months she complained of persistent wrist pain. The fracture had been previously considered radiologically healed, and she was doing daily routine activities. Her hematological parameters were within normal limits. On MRI examination, there was a 3 x 4 mm focal lesion in the lateral part of the lunate, showing: hypointensity on T1-weighted images and hyperintensity on fat-suppressed sequences (Figure 2). These findings were suggestive of early focal avascular necrosis (AVN) of lunate, with possible residual viable bone. No carpal malalignment was identified. No joint disease was detected. No adjacent soft tissue abnormality was detected. A CT scan was not carried out due to lack of clinical indication and radiation concerns.

**Figure 3:**
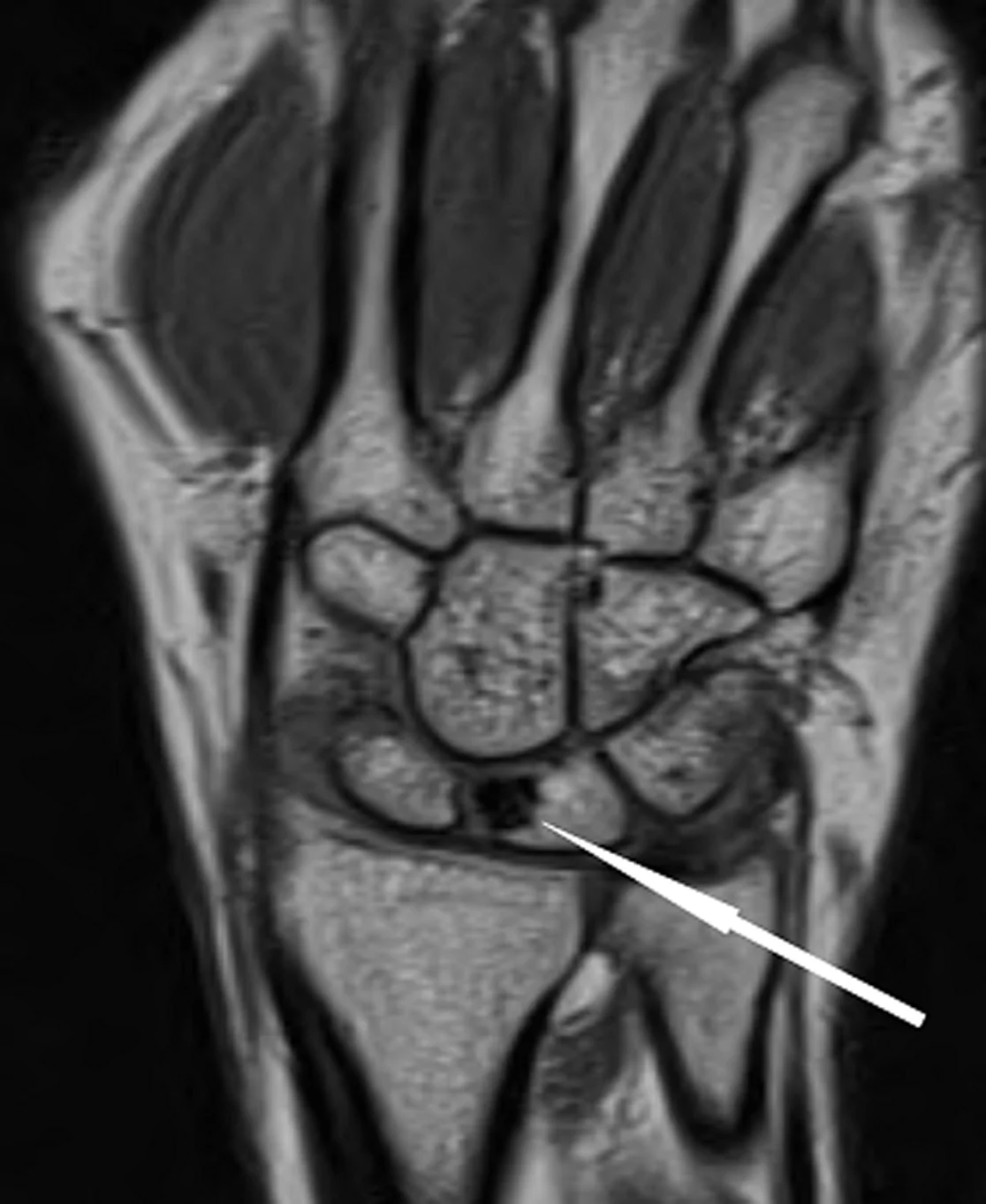
coronal T1-weighted MRI of the wrist demonstrates a focal hypointense area in the lunate (arrow), consistent with chronic avascular necrosis

**Case 3:** a 49-year-old male presented with a history of a left wrist lunate fracture a few years prior. He was managed conservatively. For the last few months, he complained of mild wrist pain. The fracture had been previously considered radiologically healed, and he was doing day-to-day activities. His hematological parameters were within normal limits. Magnetic resonance imaging of the wrist was performed for further evaluation, which showed a 5 x 4 mm focal lesion in the lateral part of the lunate, hypointense both in T1-weighted images (Figure 3) and fat-suppressed sequences. These findings were suggestive of chronic focal avascular necrosis (AVN) of lunate bone. No carpal malalignment was identified. No joint disease was detected, and no abnormal signal changes were seen in adjacent soft tissue. A CT scan was also not carried out in this patient due to lack of clinical indication and radiation concerns.

## Discussion

In our series of three patients, MRI consistently demonstrated features of avascular necrosis despite prior imaging suggesting fracture union. The CT-MRI discrepancy shows the limitation of structural imaging in symptomatic patients following wrist trauma. In all three cases, prior trauma might have led to disruption of the normal pattern of bone vascularity, which predisposed to ischemic changes. This is consistent with the known susceptibility of carpal bones to post-traumatic vascular compromise [1,2,4,9]. In Case 1, MRI demonstrated abnormal signal in the proximal pole of the scaphoid, whereas CT showed apparent fracture union with mild sclerosis and no evidence of collapse. These findings suggest that, despite structural healing, early ischemic changes may still be present. Similar discordance between CT and MRI findings has been described previously [7,8].

Magnetic resonance imaging is useful for detecting early changes in bone viability, as it can identify subtle alterations in marrow signal [6,7]. In our series, the affected bones showed T1 hypointensity with corresponding hyperintensity on fluid-sensitive sequences in two cases, consistent with early ischemic change. The third case demonstrated persistent low signal, suggesting chronic disease. These findings often precede structural changes such as collapse, which may not be evident on radiographs or CT [7,10]. Persistent wrist pain following an apparently healed carpal fracture should not be overlooked. In such cases, MRI can provide additional information on bone viability that is not detected by CT alone. Early recognition of vascular compromise may help guide management and reduce the risk of progression [3,9].

In low- and middle-income settings such as India, particularly in resource-constrained regions like Odisha, access to advanced imaging modalities such as MRI may be limited by cost and availability. Consequently, patients with wrist injuries are often assessed using conventional radiography or CT. However, as demonstrated in our series, apparent fracture union on CT may not reflect underlying bone viability. Failure to detect early avascular necrosis can result in delayed diagnosis, progression to collapse, and the need for more complex and costly interventions. Therefore, in patients with persistent or unexplained wrist pain following apparent fracture healing, selective and clinically indicated use of MRI can be a cost-effective strategy, as it facilitates early diagnosis and may help avoid long-term disability and higher treatment expenses.

**Limitations:** contrast-enhanced MRI was not performed, which could have provided more definitive assessment of bone perfusion and viability. Clinical examination findings and longitudinal follow-up data were not available, limiting correlation with functional outcomes and disease progression. In cases 2 and 3, CT imaging was not performed due to lack of clinical indication and to avoid unnecessary radiation exposure.

## Conclusion

Magnetic resonance imaging can detect both early and chronic avascular necrosis in carpal bones. In our series, MRI findings directly influenced clinical suspicion and highlighted ongoing bone ischemia despite apparent structural healing on CT. Persistent or new-onset wrist pain after a healed fracture should prompt MRI evaluation to assess bone viability and guide timely management. In resource-limited settings, its judicious use in selected patients may help prevent progression to collapse and reduce long-term functional and economic burden.

### What is known about this topic

- Carpal bone fractures, particularly of the scaphoid and lunate, are associated with a risk of avascular necrosis due to their vulnerable blood supply;
- Computed tomography is commonly used to assess fracture healing but primarily reflects structural integrity rather than bone viability;
- Magnetic resonance imaging is more sensitive in detecting early marrow changes and ischemia, allowing earlier diagnosis of avascular necrosis.

### What this study adds

- This case series shows that avascular necrosis can be present despite apparent fracture union on CT, highlighting a potential diagnostic pitfall;
- It emphasizes the value of MRI in patients with persistent wrist pain following presumed fracture healing for early detection of bone ischemia;
- It highlights the importance of selective MRI use in resource-limited settings (such as parts of India and Odisha) to enable timely diagnosis and reduce long-term disability and treatment costs.
